# Tumor-Derived Lactate Creates a Favorable Niche for Tumor *via* Supplying Energy Source for Tumor and Modulating the Tumor Microenvironment

**DOI:** 10.3389/fcell.2022.808859

**Published:** 2022-05-13

**Authors:** Mengyao Jin, Wei Cao, Bo Chen, Maoming Xiong, Guodong Cao

**Affiliations:** Department of General Surgery, First Affiliated Hospital of Anhui Medical University, Hefei, China

**Keywords:** lactate, tumor micoenvironment, energy source, immune response, lactylation

## Abstract

Tumor evolution is influenced by events involving tumor cells and the environment in which they live, known as the tumor microenvironment (TME). TME is a functional and structural niche composed of tumor cells, endothelial cells (ECs), cancer-associated fibroblasts (CAFs), mesenchymal stromal cells (MSCs), and a subset of immune cells (macrophages, dendritic cells, natural killer cells, T cells, B cells). Otto Warburg revealed the Warburg effect in 1923, a characteristic metabolic mechanism of tumor cells that performs high glucose uptake and excessive lactate formation even in abundant oxygen. Tumor tissues excrete a large amount of lactate into the extracellular microenvironment in response to TME’s hypoxic or semi-hypoxic state. High lactate concentrations in tumor biopsies have been linked to metastasis and poor clinical outcome. This indicates that the metabolite may play a role in carcinogenesis and lead to immune escape in TME. Lactate is now recognized as an essential carbon source for cellular metabolism and as a signaling molecule in TME, forming an active niche that influences tumor progression. This review summarized the advanced literature demonstrating the functional role of lactate in TME remodeling, elucidating how lactate shapes the behavior and the phenotype of both tumor cells and tumor-associated cells. We also concluded the intriguing interactions of multiple immune cells in TME. Additionally, we demonstrated how lactate functioned as a novel function factor by being used in a new histone modification, histone lysine lactylation, and to regulate gene expression in TME. Ultimately, because lactate created a favorable niche for tumor progression, we summarized potential anti-tumor strategies targeting lactate metabolism and signaling to investigate better cancer treatment.

## Introduction

**FIGURE 1:**
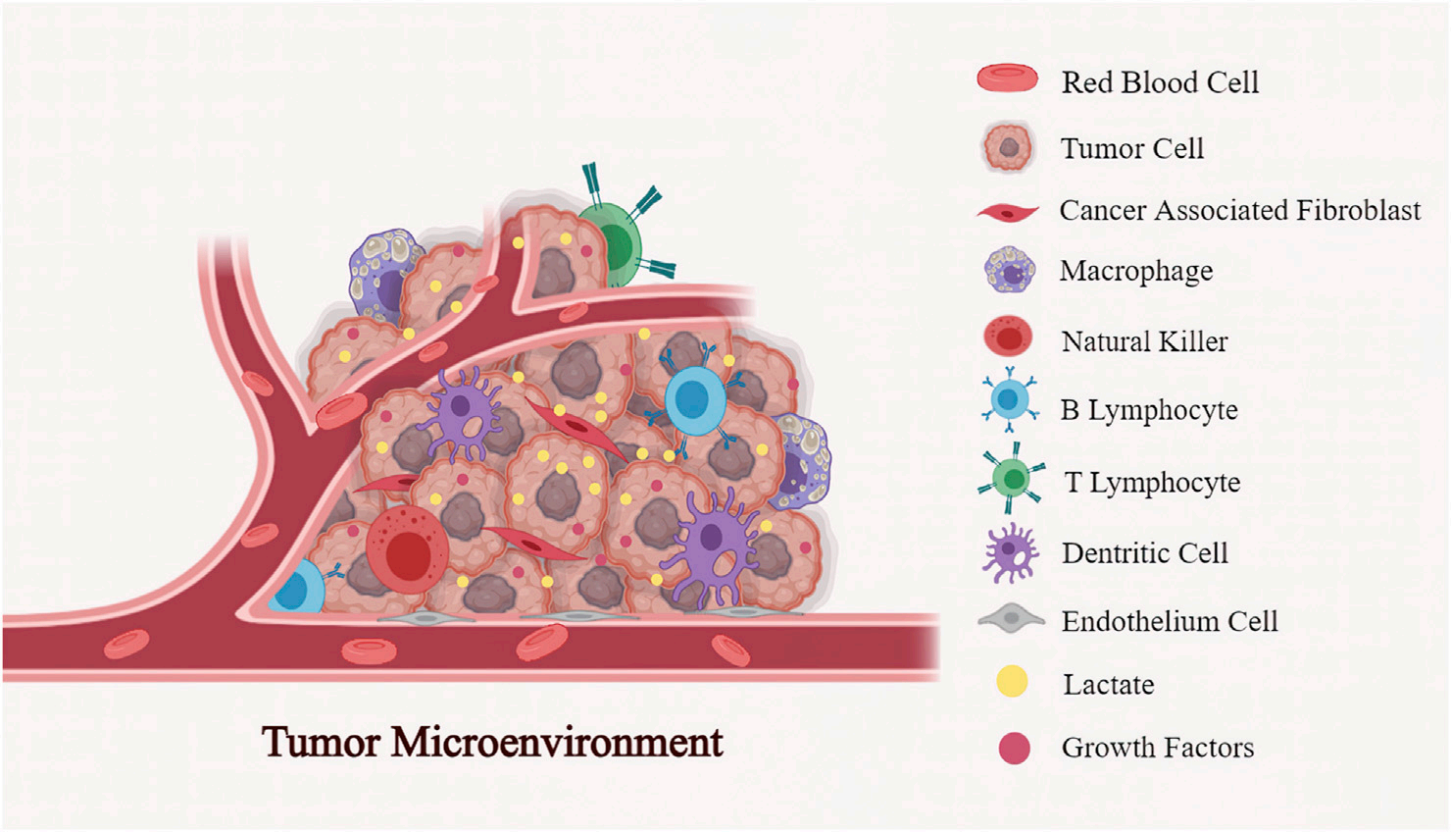
The multiple components in the tumor microenvironment (TME): TME is a functional and structural niche composed of tumor cells, the surrounding tumor stromal cells (cancer-associated fibroblast (CAFs), mesenchymal stromal cells (MSCs), endothelial cells (ECs), and immune cells (T cells, B cells, NK cells, dendritic cells, macrophages et al.). Being the primary metabilite in the tumor milieu, lactate involves in the interactions between cancer cells and stromal cells to reprogramme TME, further regulating the tumor evolution.

The tumor microenvironment (TME) is an intricate environment made up of tumor cells, blood vessels, stromal cells, endothelial cells (ECs), cellular metabolites, nutrients, and growth factors (Figure 1). Otto Warburg found in the 1920s that tumors consume massive amounts of glucose compared to surrounding tissue. Tumor cells obtain ATP through aerobic glycolysis and produce excessive lactate intracellularly even when oxygen is enough (Warburg et al., 1924; Warburg, 1925). However, they observed that tumor viability could be maintained solely through respiration. Subsequently, in 1929, an English biochemist named Herbert Crabtree extended Warburg’s work and investigated the heterogeneity of glycolysis in various tumor types (Crabtree, 1929). He confirmed Warburg’s findings but added that the magnitude of respiration in tumors varied, with many tumors exhibiting significance (Crabtree, 1929). As a result, Crabtree inferred that tumor cells exhibit aerobic glycolysis and vary in fermentation, most likely due to environmental or genetic influences. Efraim Racker coined the term “Warburg Effect” in the early 1970s.

Compared to breaking down glucose via mitochondrial oxidative phosphorylation (OXPHOS), Warburg-dependent cells use a “far less efficient” mechanism to obtain ATP. Indeed, the end-product lactate concentration was found to be significantly elevated in glycolytic tumors (1–40 mM) (Walenta et al., 2004), being the most strongly elevated metabolite in TME. Others have suggested that lactate levels are strongly linked to tumor aggressiveness and poor survival (Hui et al., 2017). Lactate is no longer just a byproduct; it is now the primary metabolite in the crosstalk of tumor and stromal cells.

## Unique Metabolism in Warburg-Dependent Tumor Cells Lead to a Reprogrammed TME

Rapid tumor cell progression leads to increased oxygen consumption and limited nutrients, resulting in a severe hypoxic and nutritionally urgent TME (Petrova et al., 2018). TME is also characterized by disorganized vascularization and extracellular acidosis (Vaupel and Mayer, 2016). TME has been remolded by the characteristic metabolic pathways of malignant proliferating cells, which influence the multiple metabolic activities in TME. Notably, the metabolite lactate has been shown to form tight couplings with other components of TME, thereby promoting tumor progression.

### Hypoxia Contributes to Remolding an Acidified Immunosuppressive TME

Under hypoxic conditions, the transcription factor hypoxia-inducible factor 1 (HIF1) is stabilized and then shifted to the nucleus to bind to hypoxia-regulated genes responsible for facilitating anaerobic glycolysis and regulating vessel generation, inducing further hypoxic responses (Vaupel, 2008). Several downstream effects of hypoxia-dependent HIF-1α activation are linked to immune escape. The immunosuppressive effects can further be classified as accumulated lactate, acidified TME, and over-expressed VEGF.

Hypoxia stimulates anaerobic glycolysis, which increases lactate generation and shuttling. Accumulated lactate can be transported into cells and used as a metabolic substrate in the hypoxic TME. The acidification in TME ranges from 6.0 to 6.5 due to the newly generated lactate exported together with H^+^ by the tumor cells. The acidic TME formed by Warburg-dependent tumor cells has been shown to influence a subset of cells, including immune cells, cancer-associated fibroblasts (CAFs), endothelial cells, and stromal cells (Faubert et al., 2017).

Hypoxia-/HIF-1α can induce VEGF expression and VEGF-R activation, further suppressing anti-tumor immune responses. Endothelial-cell activation and angiogenesis are aided by tumor-derived lactate via HIF-dependent and non-HIF-dependent pathways. Both pathways are involved in monocarboxylate transporters 1-mediated lactate transport and subsequent inhibition of prolyl hydroxylases (PHDs) (Sonveaux et al., 2012; Rivera and Bergers, 2015; Rivera et al., 2015; Horikawa et al., 2017). Mechanistic studies showed that M2-like macrophages, Treg cells, and certain inhibitory molecules such as PD-L1 were involved in mediating the activities (Vaupel and Multhoff, 2016).

### Lactate Establishes Intricate Couplings Between Metabolic and Genetic Variations Within the Reprogrammed TME

According to the existing literature, high lactate concentrations (median concentrations >8 mmol/L) were related to subsequent metastasis in malignant tumors (Marchiq and Pouysségur, 2016). Even though regarded as a “metabolic waste product” for a long period, lactate has now been widely acknowledged as a source of metabolic energy and an oncometabolite with signaling properties.

Tumor cells are organized to meet increased glucose needs for multiplication in response to the excessive lactate produced by accelerated aerobic glycolysis. Tumor cells showed increased glucose uptake, decreased mitochondrial function, upregulated monocarboxylate transporters (MCT), and glycolytic enzyme expression. According to published research, lactate in TME can be utilized as a secondary energy source by tumor cells and then extruded to nearby endothelial and stromal cells (Lyssiotis and Kimmelman, 2017).

Furthermore, lactate promotes tight metabolic connections between tumor cells, stromal cells, and immune cells. Lactate can facilitate immune evasion of lymphocyte identification and dampen the efficacy of anti-tumor therapy in the programmed TME. Lactate appears essential for tumor evolution by acting as a messenger between the producer and consumer cells in TME. Moreover, lactate is an essential metabolite in transcriptional regulation. Zhang et al. (2019) revealed a previously unknown histone modification in which lactate confers specific gene expression signatures in M1 macrophages through substrate provision, now known as lysine lactylation.

Lactate has been elucidated to participate in angiogenesis, energy supply, immunosuppression, and epigenetic alterations. These intricate couplings between metabolic and genetic variations in the reprogrammed TME have opened up new avenues for further treatment strategies. Thus, it is critical to explore the origin and location of lactate before determining its vital role in TME.

## Lactate Accumulation and Shuttling in TME

### Characteristic Glycolysis and Glutaminolysis Metabolic Pathways Lead to Excessive Lactate

Lactate is a hydroxycarboxylic acid broken down in the human body into D-lactate and L-lactate. L-lactate is the main physiological enantiomer of lactate, while D-lactate accounts for only 1%–5% of the latter (Connor et al., 2017). Glucose is partially oxidized into pyruvate, reduced to lactate, and extruded extracellular in Warburg-dependent tumors. Besides that, glutamine catabolism produces tumor-derived lactate. One of these routes is the conversion of glutamine-derived carbon to heraloacetyl in the citrate cycle to malate, which then exits the mitochondria and is converted to NADPH and pyruvate in the cytoplasm by malaise. The primary carbon source for lactate generation in tumor cells is glucose exploitation via glycolysis, with glutamine breakdown via glutaminolysis serving as a secondary but significant source. The characteristic glycolysis process results in an accumulation of lactate in the cytosol and an excess of H^+^. The main populations responsible for lactate production in TME were tumor cells and cancer-associated fibroblasts (CAFs). The inefficiency of glycolysis and the urgent need for ATP results in significantly increased glucose uptake, which results in accumulated intermediates substances such as lactate.

The solid tumors and cancer cells maintain extracellular acidity because of high lactate production. To sustain metabolism, the tumor cell must expel lactate from the cell. Excessive cytosolic lactate has been shown to effectively reduce the glycolytic rate by inhibiting the rate-limiting enzyme phosphofructokinase-1 (PFK-1). Accumulated lactate facilitates the conversion of lactate into pyruvate mediated by lactate dehydrogenase (LDH), resulting high amount of NADH generation and subsequent inhibitory feedback on glycolysis (Trivedi and Danforth, 2016). To maintain high-rate glycolysis, eukaryotic cells must drive lactate and H^+^ efflux to extracellular space to avoid intracellular acidification (Pucino et al., 2019). Emerging evidence has shown that proton-coupled lactate efflux from tumor cells or stromal cells is important in preserving the acidic phenotype and promoting tumor dissemination by remolding the TME, resulting in angiogenesis and cell invasion metastasis, and immune escape (Ippolito et al., 2019). Lactate shuttle in rat skeletal muscle is driven by a concentration and pH gradient or the cellular redox state.

Nonetheless, there is a remarkable increase in lactate levels (1–40 mM) in glycolytic tumors, closely related to tumor aggressiveness and poor prognosis. Rapid shuttle appears to be at odds with lactate accumulation in tumors. The net increase in lactate concentrations within the tumor, on the other hand, could be explained by the higher glucose-to-lactate flux (which characterizes the Warburg-dependent cells) versus the lactate-to-CO_2_ flux in lactate-dependent cells. It could also be explained by the abundance of Warburg-dependent cells in a growing tumor, a feature that can be measured in clinical practice with fluorodeoxyglucose (FDG)–positron emission tomography (PET) to monitor tumor progression.

### Lactate Dehydrogenase : A Tetrameric Enzyme Crucial for Lactate Synthesis

Many studies have found that lactate metabolic coupling is based on the reversible reaction of the nicotinamide adenine dinucleotide (NAD+) oxidoreductase LDH enzyme. This tetrameric enzyme is composed of M and H protein subunits encoded by the LDHA and LDHB genes. The genes can assemble in five different heterotetramers or homotetramers in a tissue-dependent manner. There are five isoenzyme formations: LDH-1 (4H), LDH-2 (3H1M), LDH-3 (2H2M), LDH-4 (1H3M), and LDH-5 (4M). LDH is typically found within the cell, and the isoenzyme composition varies between tissues. The LDHA isoform is mainly expressed in skeletal muscle. It preferentially converts pyruvate to lactate, whereas the LDHB isoform is located commonly in the heart and brain and preferentially converts lactate to pyruvate (Markert et al., 1975). LDH is the primary metabolic enzyme responsible for converting pyruvate to lactate and vice versa. LDH is required to regulate nutrient exchange between tumors and the stroma.

Increased LDH expression and activity have been observed in various tumor types is related to chemoresistance and a low event-free survival rate. High LDHA levels in serum can be regarded as a negative prognostic biomarker in malignancies (Zhang et al., 2019a), indicating that a large amount of lactate is secreted from tumor cells into the circulatory system. Inside tumor cells, LDHA can prevent pyruvate from entering into the mitochondrial tricarboxylic acid cycle (Ždralevi´c et al., 2018) and promote the rapid conversion of pyruvate to lactate (Jiang et al., 2016). High-level LDHA promotes tumor cell formation and progression by facilitating epithelial to mesenchymal transformation (Arseneault et al., 2013), angiogenesis, cytoskeletal remodeling (Valvona and Fillmore, 2018), cell invasion, and migration (Liu et al., 2015).

Similarly, LDHB expression may be used as a biomarker for therapy response in various cancers. For instance, LDHB expression could be used to assess the efficacy of neoadjuvant chemotherapy in breast cancers. Cancer cells with glycolytic and base-like phenotypes were found to have high LDHB expression, whereas LDHB knockdown reduced glycolytic dependence. Patients with basal-like cancers had high levels of LDHB expression and had a complete pathological response (pCR) to neoadjuvant chemotherapy (Dennison et al., 2013). A study by Ždralevi´c elucidated that LDHA/LDHB double knockout (LDHA/B-DKO) completely suppressed glycolysis, whereas LDHA or LDHB gene knockout alone failed to inhibit lactate production. LDHA/B-DKO completely stopped cell growth because they could not switch metabolism to oxidative phosphorylation (OXPHOS) in a hypoxic environment (Ždralevi´c et al., 2018).

In conclusion, elevated LDH expression is linked to poor prognosis in tumor patients. LDH regulates lactate production, which is important in tumor progression (Certo et al., 2019). In various tumor entities, a positive correlation between LDH, high lactate levels, and tumor progression has been documented (Girgis et al., 2014)^,^ implying that targeting human LDH may be beneficial for treating advanced cancers.

### Monocarboxylate Transporter: A Plasma Membrane Transporter Vital for Lactate Shuttling in Bulk Tumors

Under physiological pH, lactate can be completely dissociated into lactate anion, which cannot pass through the plasma membrane via free diffusion. The transport mechanism relies on proton-like MCTs (Garcia et al., 1994; Jones and Morris, 2016). MCTs comprise four reversible types (classical H+/lactate symporters) from the SLC16/MCT family of solute carriers, consisting of 14 members with conserved sequence motifs (Renner et al., 2017). MCT1 and MCT4 are present in monocytes, lymphocytes, and granulocytes (Merezhinskaya et al., 2004). Furthermore, MCT1 (SLC16A1) and MCT4 (SLC16A3) are ubiquitously expressed in the human body, with an obvious up-regulation observed in malignant tumors.

Over-expression of MCTs has been shown to validly moderate the stress caused by accelerated lactate generation in tumor cells *via* adjustable bidirectional transport depending on the TME and cellular context. MCTs1 first bind to a free proton, followed by lactate binding and a conformational change, which then mediates lactate extrusion to the opposite side of the membrane. At the end of the transport phase, the proton would be released. In normal tissues, high-affinity MCT1 is the primary transporter responsible for lactate homeostasis, as both input and output correlate with the lactate transmembrane gradient. However, in anaerobic glycolysis tumor cells, the accumulated lactate would quickly saturate MCT1 so that tumor cells always rely on the low-affinity MCT4 to accomplish lactate export instead. Lactate released by lactate biosynthetic addicted cells through MCTs can be utilized by several cells as an energy-rich byproduct, which MCT1 mainly uploads.

## Lactate Serves as a Secondary Energy Source in TME: A Metabolic Symbiosis

Rapid-growing solid tumors, characterized by rapid proliferation and high energy consumption, are generally nutrient-deficient, aggravated by insufficient vascular supply. As tumor proliferates faster than vascularization, only tumor cells near the vessels acquire oxygen and thus remain normoxic. In contrast, tumor cells have insufficient oxygen supply and thus remain hypoxic. To adapt to this complex, stressed environment, metabolic genes have remolded the TME to meet the demand for proliferation (Baek et al., 2014). In the TME, there are normoxic and hypoxic cell populations, with the latter naturally lacking the ability to oxidize lactate. In the TME, a symbiotic relationship in lactate metabolism has been observed between normoxic and hypoxic cells. Several studies have illustrated that within the TME, a subset of cells undergoes Warburg-like metabolism, while another subset decomposes the lactate via OXPHOS-dependent metabolism (Faubert et al., 2017; Hui et al., 2017).

**FIGURE 2:**
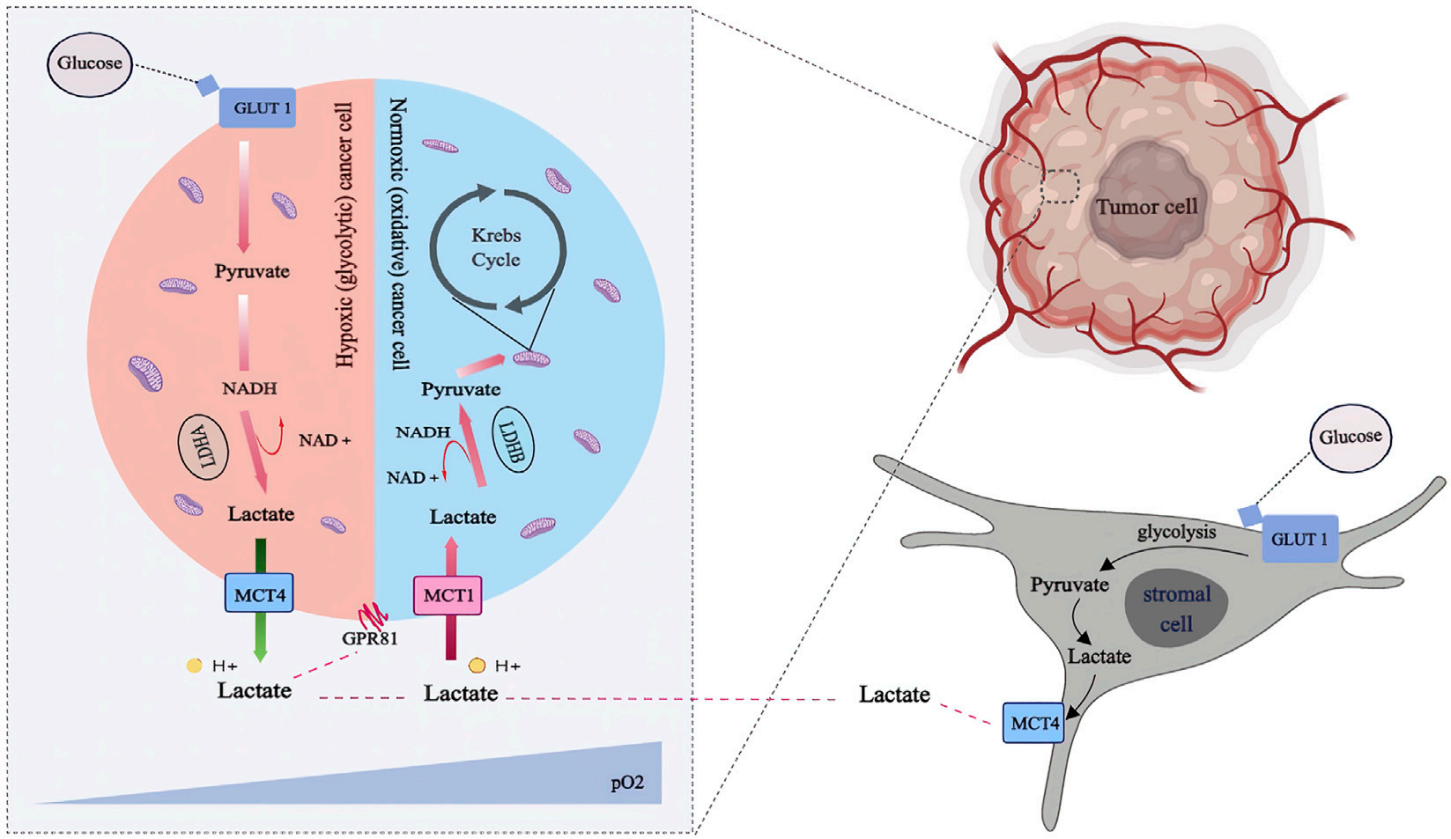
Lactate shuttling in the metabolic symbiosis: Lactate released through the monocarboxylate transporter. Tumor cells with profuse oxygen supply (pale blue) preferentially utilize lactate as an oxidative fuel, which spare glucose such that make it available for both glycolytic tumor cells (pale blue) and stromal cells (pale gray), via anaerobic and aerobic glycolysis, respectively. Hypoxic tumor cells and stromal cells generate lactate preferentially to fuel canonical lactate biosynthesis via Lactate Dehydrogenases (LDH) isoform A and export the lactate out of the cells via MCT4. Then the lactate can be transported into normoxic tumor cells via MCT1 and subsequently transformed into pyruvate via LDHB for ATP production.

A favorable location with abundant nutrition substance and the ability to establish a metabolic symbiosis with hypoxic cancer cells maintains either oxygenated tumor cells or nearby vessels (Figure 2). Both metabolic characteristics and hypoxia-inducible factor-1α (HIF-1α) expression were significantly different in the cell populations. Moreover, metabolic symbiosis occurs between different cancer cell populations within the tumor and between normoxic cancer cells and tumor-associated stromal cells, particularly fibroblasts. Cancer-associated fibroblasts (CAFs) and tumor cells are the major cell types with mesenchymal-like characteristics in solid tumors, supporting cancer cells by providing additional paracrine factor nutrients and supplementing the nutrient reserves provided by local vessels (Brand et al., 2016). Stromal cells are activated and transformed into tumor-associated stromal cells (TASCs) in the reprogrammed TME, where they play a role in modulating the cancer phenotype. In the reprogrammed TME, tumor cells induce fibroblasts to switch to an “aerobic glycolysis” metabolic mode and produce lactate. The “instructed” fibroblasts lack caveolin-1 but are abundant in oxidant radicals, TGF-β and HIF-1α, possessing a suitable environment to program enzymatic pathways relevant to “aerobic glycolysis” and subsequent lactate production.

Essentially, tumor cells with abundant oxygen supply preferentially use lactate as an oxidative fuel, sparing glucose and making it available for glycolytic tumor cells and stromal cells via anaerobic and aerobic glycolysis, respectively. Hypoxic tumor cells and stromal cells utilize glucose at a high rate, resulting in excess lactate. On the one hand, hypoxic tumor cells and stromal cells preferentially generate lactate to fuel canonical lactate biosynthesis through lactate dehydrogenase (LDH) isoform A and export the lactate out of the cells via MCT4. On the other hand, Normoxic tumor cells upload this lactate by MCT1 to be converted into pyruvate for ATP production via LDHB. LDHB expression can be triggered by an increase in lactate concentration in TME among neighboring stromal cells such as CAFs. The influxed lactate is converted to pyruvate with the help of LDHB present in CAFs, which is then used as a valuable fuel for the function of CAFs (Shi et al., 2017) and also utilized by cancer cells by a reciprocally-supportive metabolic relationship (Rattigan et al., 2012). This correlative signaling promotes tumor growth by fostering positive feedback loops. LDHB is essential in the stromal-metabolic reprogrammed TME mediated by cancer, and it serves as the foundation for the stromal-epithelial metabolic coupling pathway (Patel et al., 2017).

Mechanistic studies demonstrate that lactate is produced by aerobic glycolysis, which is maintained by SIRT3/succinate-dependent-HIF-1α activation, eventually leading to a reaction catalyzed by LDHA. LDHB can decompose lactate into pyruvate, which can be diverted into the tricarboxylic acid (TCA) cycle to provide energy. Furthermore, it directly binds to oxygen sensors such as N-Myc downstream-regulated gene 3 (NDRG3), thereby modulating redox state and lysosomal function via the LDH reaction. Furthermore, the binding process could stabilize HIF-1, induce reactive oxygen species (ROS), and activate nuclear factor-kB (NF-kB) signaling, thereby increasing transcriptions of genes encoding cytokines such as hepatocyte growth factor (HGF), interleukin-8 (IL-8), and vascular endothelial growth factor (VEGF). Lactate can be sensed extracellularly by G protein-coupled receptor 81 (GPR81), inducing signal transduction and facilitating intracellular lactate exploitation.

## Histone Lactylation: A Novel Post-Translational Modification Established by Lactate

Otto Warburg’s observations in the 1950s highlighted preferential production of lactate by glycolysis even in the absence of oxygen. Cancer cells rewire metabolism to promote glucose uptake and breakdown, allowing for the rapid synthesis of energy and biosynthetic precursors required to produce a progeny cell. According to new research, the end-product lactate is no longer a waste metabolite of proliferating tumor cells. Still, it acts as an energy source, a signaling molecule, and an immunoregulatory molecule. Although the end-product lactate has been extensively explored, with numerous reports on its role in TME restructuring, its precise contribution to cellular function remains unknown.

### Histone Modification

A dynamic balance between the enzymatic activities of writers and erasers modulates histones Kac. Non-enzymatic lysine acylation is also thought to occur, particularly in mitochondria, where relatively high concentrations of pH and acyl-CoA would benefit them (Moellering and Cravatt, 2013; Wagner and Payne, 2013; Gouirand et al., 2018). On the other hand, conditions in the nucleus are less favorable to these processes (Weinert et al., 2015). Instead, previously characterized histone acetyltransferases (HATs) were shown to have extended acyltransferase activities. HATs consist of three families of sequence and structural features; GNAT (Gcn5-related N-acetyltransferase), the p300/CREB binding protein (p300/CBP), and MYST (Moz, Ybf2, Sas2, and Tip60) (Lee and Workman, 2007; Weinert et al., 2014). All three families of HATs have been shown to use a variety of acyl-CoA as substrates for histone Lys acylation. HATp300 (also known as EP300), a well-studied transcriptional coactivator, has emerged as the most confounding acyltransferase identified to date. Structural studies of p300 reveal a deep aliphatic pocket within the active site, a missing feature in GCN5 and other HATs like TIP60 and MOF (also known as KAT8) (Jing and Lin, 2015).

Zhang et al. (2019) from the University of Chicago established a novel function for lactate whereby it confers specific gene expression signatures in M1 macrophages by providing substrate for a previously unknown histone modification, now known as lysine lactylation (Zhang et al., 2019b).

### Histone Lactylation Reacts to Regulate Downstream Gene Expression

**FIGURE 3:**
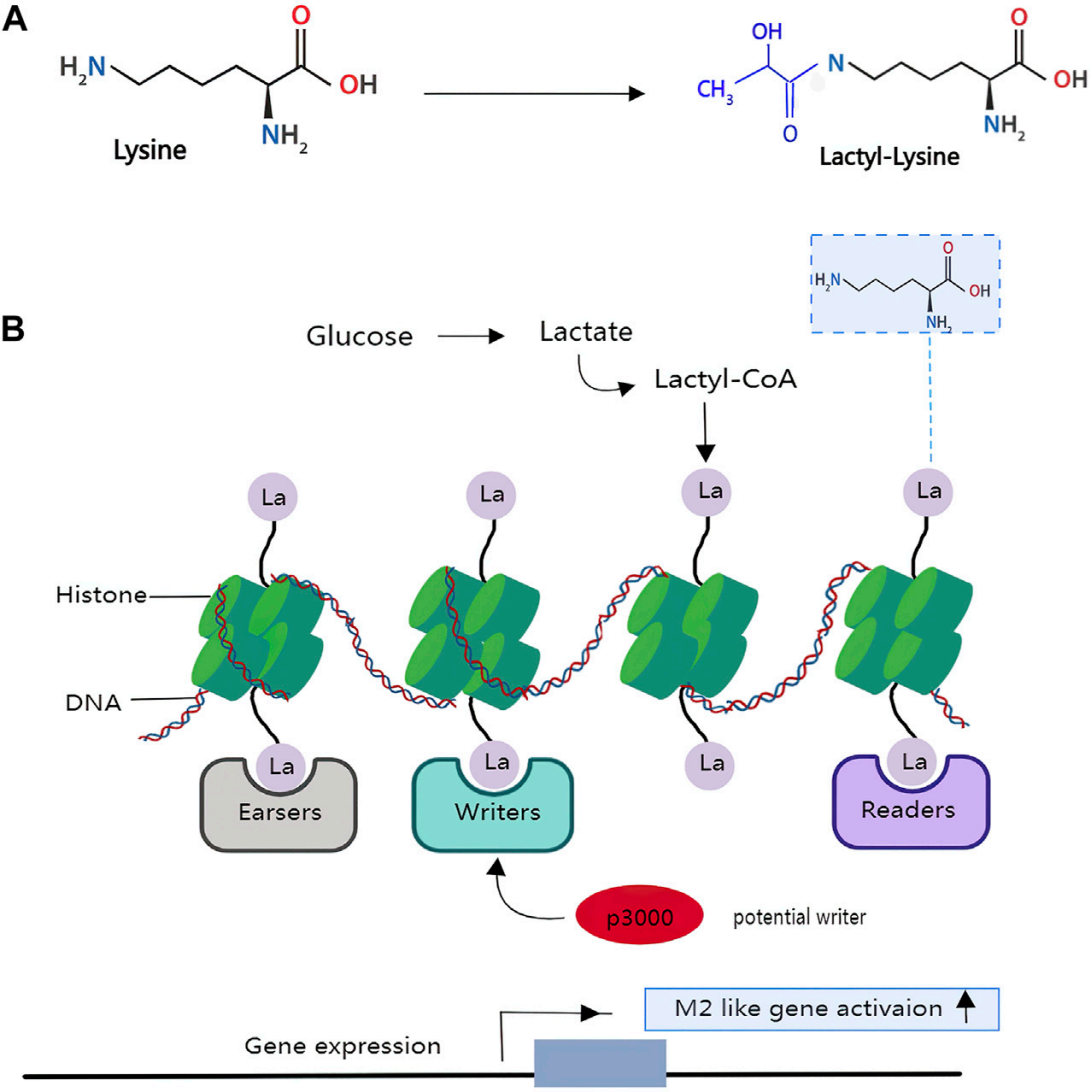
Lactate modifies histones to regulate macrophage polarization and tumour immunity: An achemical modification called lactylation—the addition of a lactyl (La) group to the lysine amino-acid residues in the tails of histone proteins. **(A)**, the structure of lysine and lactyl group. **(B)**, glucose can be incompletely converted to the metabolite lactate in the macrophages or under a hypoxia situation. The lactyl-CoA generated from lactate contributes a lactyl group to the lysine tails of histone proteins via the acetyltransferase enzyme p300 to produce the epigenetic modification called lactyllysine, which allows for gene activation of genes belonging to wound-healing pathways, thus resulting in an M2-like phenotype. However, it is unclear which enzymes generate the intermediate molecule lactyl-CoA, from which La is derived, or which enzymes deposit (writers), remove (erasers) or recognize and interpret (readers) histone lactylation.

Histones are a type of protein that consists of a nucleosome core wrapped in DNA. Histones are influenced by several post-translational modifications (PTMs) that characterize and form functional chromatin states. A unique mass shift of 72 Da was detected on lysine residues of histone tails, extracted from a human cancer cell line using mass spectrometry (Huang et al., 2014). The authors hypothesize that the mass shift is caused by the addition of a lactyl group (la) to a lysine residue (K) (Figure 3). The hypothesis was confirmed when the spectrum of biochemically synthesized histone peptides with Kla modifications completely overlapped with the previously generated histone peptides. ^13^C-labeled lactyl groups are found on histone lysine residues when isotopically ^13^C-labeled lactate is used to track glycolytic metabolite. Therefore, it is reasonable to assume that histone lactylation occurs as a post-translational modification of histone *in vivo* (Zhang et al., 2019b). So far, 246 histone sites bearing these new Lys acylations have been identified.

Moreover, it has been demonstrated that histone lactylation is caused by glycolysis because tracing of isotopically labeled glucose causes the deposition of ^13^C-labeled atoms on histones. The majority of novel histone PTMs are now defined as short-chain Lys acylations. These modifications are similar to lysine acetylation (Kac), a well-studied lysine modification, but they differ in hydrocarbon chain length, hydrophobicity and charge.

Drug-mediated inhibition and promotion of glycolysis resulted in a decrease and increase in overall Kla numbers, respectively, reflecting the sensitivity of histone lactylation to the amount of lactate produced by glycolysis. Existing studies suggest that the functional and genetic targets of histone lactylation mainly lay on macrophages. Macrophages are immune cells that can be composed of two classes: a pro-inflammatory class (termed M1) and an anti-inflammatory, wound-healing class (termed M2). When there are infections in the human body, macrophages play a key role in host defense against infections through promoting tissue remodeling and clearance of cell debris. M1 macrophages rely primarily on aerobic glycolysis, which directly results in high lactate production, whereas M2 macrophages increase oxidative phosphorylation and fatty acid oxidation (Galván-Peña and O’Neill, 2014). Lactate production played a role in determining macrophage epigenetic phenotype. By conducting a genome-wide, unbiased approach, the authors observed that lysine lactylation is high in the promoter regions of genes which were responsible for wound healing, an M2-like phenotype. At the same time, chemically inhibition of lactate generation during M1 polarization led to reduced lactate and histone Kla levels but had no influence on the expression of pro-inflammatory genes. Thus, we deduce that histone modification is possibly acting to regulate the expression of genes.

According to emerging evidence, these modifications affect gene expression by altering the physical accessibility of the DNA molecule to proteins involved in DNA transcription. Post-translational modifications of histones are critical for maintaining homeostasis by regulating DNA-dependent processes such as transcription, replication, and DNA repair, altering nucleosome contact among themselves, and recruiting non-histone proteins (Kouzarides, 2007; Tessarz and Kouzarides, 2014). Lactate produced by glycolysis under hypoxic conditions or during a bacterial challenge has been shown to stimulate histone lactylation and thus activate downstream gene expression. In addition, histone lactylation functions act as an important epigenetic regulator during pathogenesis. When confronted with a bacterial infection, macrophages must rapidly switch to aerobic glycolysis to facilitate M1 polarization and pro-inflammatory cytokine production. Once the infection has been eradicated, macrophages must ensure that the inflammatory response is dampened to avoid collateral damage. Moreover, histone lactylation at pluripotency gene loci induced by Glis1 benefited somatic cell reprogramming. These findings highlighted the profound impacts of individual metabolites on cellular function (Pucino et al., 2017).

### A Bridge Exists Between Histone Modifications and RNA Modifications

The balance between transcription activation and repression may be disrupted by dysregulation of histone modifications, which is linked to many diseases, including developmental and neurological disorders, as well as various cancer aetiologies (Lewis et al., 2013; Ronan et al., 2013; Pavlova and Thompson, 2016). For instance, an association study of the entire histone acetylation group identified 4,162 distinct H3K27ac peaks enriched in disease-associated biological pathways between Alzheimer’s disease cases and controls (Marzi et al., 2018). Furthermore, H3K9me3 demethylation mediated by KDM4A in oocytes is necessary for normal activation of the zygotic genome and preimplant development after fertilization, whereas absence of KDM4A leads to insufficient transcriptional activation of the genes (Sankar et al., 2020). Above all, histone modifications are multiple markers tightly correlated with the occurrence and development of disease, and exploring the role of histone modifications in disease pathogenesis, especially tumorigenesis has gradually become a research hotspot. Yu et al. (2021) firstly illustrated that histone lactylation was increased in tumors and is correlated with poor prognosis in ocular melanoma (Yu et al., 2021). Target correction of abnormal histone lactylation triggers therapeutic efficacy both *in vitro* and *in vivo*. Histone lactylation promotes the transcription of YTHN6-methyladenosine RNA-binding protein 2 (YTHDF2), which recognizes the m^6^A modification site on RNA of two tumor suppressor genes (PER1 and TP53) and accelerates their degradation.

The novel finding of short-chain acylations on histone lysines has validly increased the complexity of histone PTMs and their interplay with cellular metabolism. Zhang et al. have provided unique insights into how lactate functions as an essential metabolite in transcriptional regulation. A few theories aimed at the regulation and function of histone acylations have also emerged. Deeper explorations of the writers, erasers, and readers, and the influence of acyl-CoA metabolism on these proteins, will help us comprehend the regulation and function of differential histone acylation. At the moment, Yu et al. (2021) have revealed the oncogenic role of histone lactylation, providing novel therapeutic targets for ocular melanoma therapy. The link between histone modifications and RNA modifications has been established, furnishing a new sight for epigenetic regulation in carcinogenesis.

## Immunosuppressive Role of Lactate in Cellular Metabolism

**FIGURE 4:**
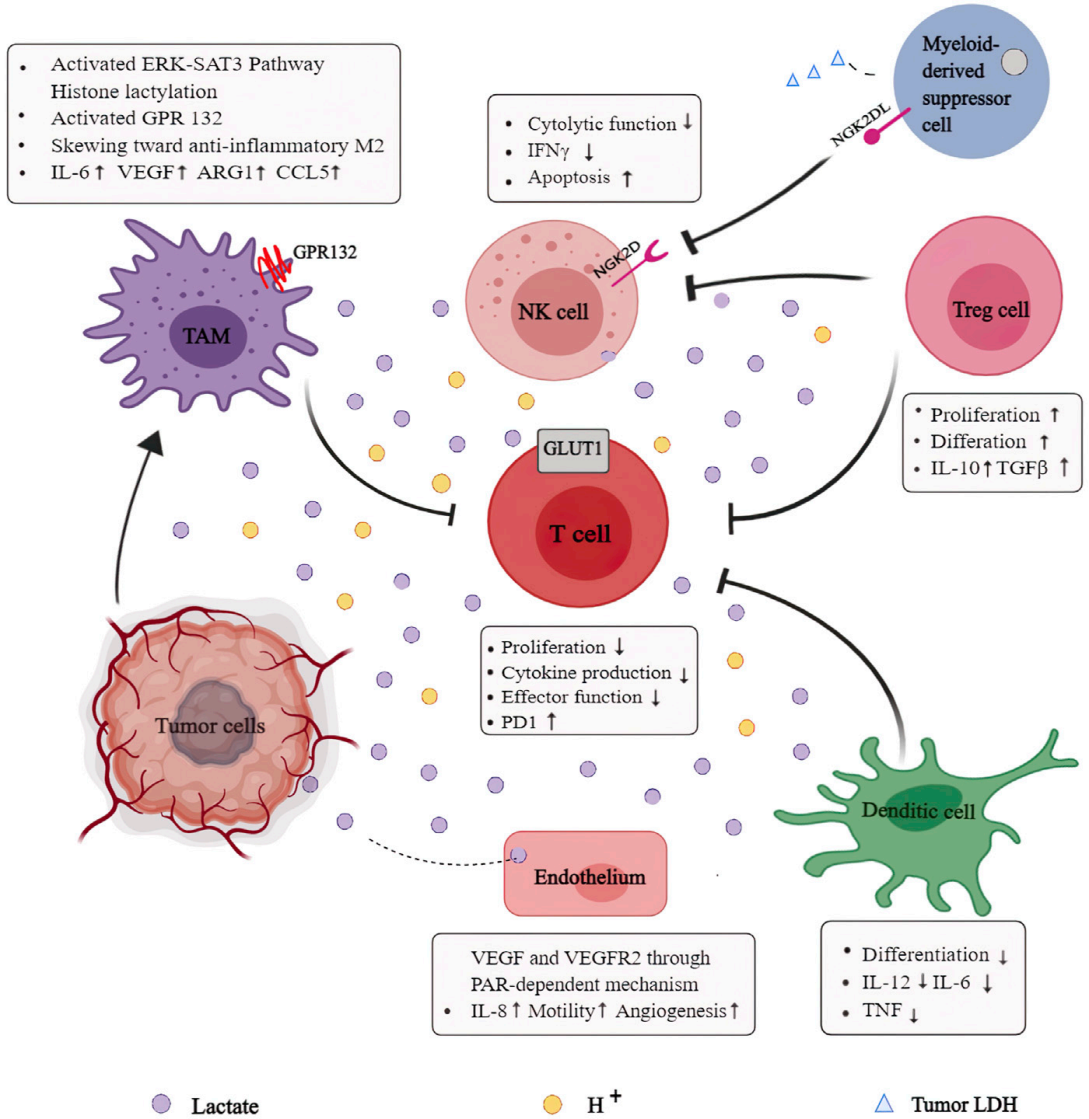
Role of lactate in the reprogrammed immunosuppressive tumor microenvironment (TME): Extracellular lactate concentrations can be perceived and endocytotic by multiple cell types, including dendritic cells, T cells, macrophages, and NK cells, to trigger intracellular signaling, modulate cell behavior, and strongly influence their function in the TME. Lactate provides immuno-metabolic coupling between tumor cells and other stromal cells, contributing to blunt immune-surveillance.

Extracellular lactate concentrations can be perceived and endocytosed by multiple cells, including dendritic cells, T cells, macrophages, and NK cells, triggering intracellular signaling and modulating cell function in TME (Figure 4). Alterations in the tumor cell signaling pathways contribute to a suppressive TME rich in inhibitory cells, posing a major obstacle to tumor immunity. Mechanistic studies have indicated that tumors can escape immune surveillance by relying on lactate metabolites and using H^+^-dependent mechanisms in an acidic environment mediated by lactate. Notably, both the lactate-dependent and H^+^-dependent mechanisms reflect the vital role that lactate plays in TME remodeling. Furthermore, the accelerated glycolysis rate induced by multiple factors such as hypoxia and accumulated lactate impairs anti-tumor responses of immune cells. Increasingly, studies focus on the interactions between lactate and multiple immune cells in TME to improve the efficacy of current anti-tumor immunotherapy.

### Lactate and Dendritic Cells

Dendritic cells (DCs) are important immune cells for initiating primary immune responses and have anti-tumor activity (Banchereau and Steinman, 1998; Bell et al., 1999). However, both circulating and tumor-infiltrating DCs of malignant tumor patients were phenotypically and functionally defective (Almand et al., 2000; Orsini et al., 2003). High lactate concentrations in TME have been certified to help dendritic cells mature, differentiate, and express antigens. Gottfried et al., in 2014 found that lactate altered antigen phenotype and functional activity of DCs modulated a specific tumor-associated DC phenotype by observing DCs differentiation in a 3-dimensional tumor model (Sutherland, 1988). The differentiated DCs were observed to have low CD1a and IL-12 *in vitro*. In addition, lactate prevents monocytes from differentiating into DCs, implying that high lactate levels in TME may impede DCs maturation.

### Lactate and T Cell

Lactate accumulation in TME is always accompanied by H^+^ accumulation, both of which can blunt T cell responses towards solid tumors. Immune checkpoint blockade (e.g., anti-PD-1, anti-PD-L1, and anti-CTLA-4) made significant progress in amplifying endogenous anti-tumor T cell responses. Besides, similar to tumor cells, naive T cells are transformed from an OXPHOS-predominant metabolism to a Warburg-dependent glycolytic metabolism after activation to meet biosynthetic and energetic demands (Chang et al., 2015). Thus, tumor cells and activated T-cells compete for glucose, which is considered a driver of cancer progression (MacIver et al., 2008).

Both T cells and cancer cells extrude lactate via MCTs to avoid intracellular acidification. Excessive lactate alters the transmembrane concentration gradient, inhibiting lactate released from activated T cells. Studies revealed that the opposing lactate efflux inhibits pro-inflammatory cytokine production and T cell cytotoxic activity by inhibiting mTORC1 (Balgi et al., 2011). Extracellular acidosis impairs T cell-mediated immunity, and neutralization of tumor acidity has been shown to improve anti-tumor responses towards immunotherapy (El-Kenawi et al., 2015). Lactate hinders the activation of the nuclear factor of activated T cells (NFAT) in NK, which is required for IFNγ transcription. Recent research has revealed that lactic acidosis suppresses JNK/c-Jun and P38 activation, impairing the function of CD8^+^ T lymphocytes (CTLs). The activation of P38 and JNK/c-JUN mediated by downstream phosphorylation of TCR-signaling is essential for IFN-γ production. Lactate has also been proven to inhibit the FAK family-interacting protein of 200 kDa (FIP200) for mediating T cell apoptosis without altering mRNA level.

Moreover, anti-tumor responses of T cells are severely compromised by complex mechanisms (Mah and Cooper, 2016), especially in tumors with a high glycolysis rate. We know that cytotoxic T cells and many effector T cells rely on glycolysis to sustain cell proliferation and cytokine production. They become inactive in insufficient glucose and excessive lactate (Bogunovic et al., 2009). Chang et al. (2015) demonstrated that increased glucose consumption inhibits T cell metabolism by lowering glycolytic capacity, mTOR activity, and IFNγproduction. Evidence suggests that glucose deprivation restrains T cell’s anti-tumor effects (Cascone et al., 2018), and the competition in the TME interferes with antigen-specific responses of tumor-infiltrating T cells.

On the other hand, lactate treatment weakens effector T cell function without meddling with Treg cell function because Treg cells obtain energy via oxidative phosphorylation (Macintyre et al., 2014) instead of glycolysis. Lactate positively affects the metabolic profile of CD4^+^ CD25^+^ regulatory T (Treg) cells, allowing them to remain in the acidic TME and amplifying their immunosuppressive functions. As the Treg-specific transcription factor, FoxP3 inhibits cMyc signaling. Subsequently, it transfers Tregs to an OXPHOS metabolism, immune-tolerant T-regulatory cells (Tregs) may be able to remain active in the TME.

### Lactate and NK Cell

Natural killer (NK) cells are a valuable target in tumor immunotherapy because they can effectively eliminate tumor cells through various mechanisms without prior sensitization. Tumor and other immune cells in the immune-suppressive TME create favorable conditions for tumor proliferation while preventing NK activation (Di Vito et al., 2019; Habif et al., 2019; Nayyar et al., 2019). The balance between activating and inhibitory signals influences NK activation. Cytokines secreted by tumor and tumor-associated cells within TME, such as transforming growth factor-β (TGF-β), IL-6, IL-10, prostaglandin E2 (PGE2), and so on can suppress NK cell activity (Konjevi´c et al., 2019; Stojanovic et al., 2013). In addition, NK activity is regulated by signals from inhibitory receptors such as CD94/NKG2A (André et al., 2018).

It has been elucidated that the metabolite lactate and low pH can significantly dampen the cytotoxic activity of NK cells in the TME, contributing to modulate an immune-suppressed TME (Husain et al., 2013). The excessive lactate generated by glycolysis can be imported into NK through transporters (including SLC16A1 and SLC16A3), further impairing ATP production. In NK extracted from murine, Brand et al. observed that the increased uptake of lactate resulted in intracellular acidification and diminished ATP levels (Brand et al., 2016). It has been reported that lactate effectively blocks the IFNγ production following PMA/Ionomycin activation in NK. Consistently, promoted apoptosis and decreased ATP were observed in liver-resident NK treated with lactate (Harmon et al., 2019). Moreover, the extracellular acidosis would present an inhibition on mTOR signaling pathway, interfering anti-tumor effects of natural NK. On the one hand, lactate directly dampens cytotoxic function and expression of perforin, granzyme, and NKp46. On the other hand, lactate recruits monocyte-derived dendritic cells to indirectly blunt NK function.

The accelerated glycolytic rate induced by multiple factors such as hypoxia and accumulated lactate poses an obvious barrier to NK in the metabolically reprogrammed TME (Harmon et al., 2019). Cong et al. observed a decrease in glycolytic rate in NK cells and impaired cytotoxic activity and cytokine production in the lung cancer microenvironment of a murine model. An inhibitory enzyme for glycolysis, fructose-1,6-bisphosphatase (FBP1), was also found to be overexpressed. FBP1 inhibition could restore NK cell’s effector functions during tumor progression (Cong et al., 2018). Given that NK cells rely heavily on glucose metabolism to carry out effector functions, glucose restriction would completely negate their anti-tumor effects, most likely in conjunction with metabolic reprogramming.

### Lactate and Macrophage Cell

Emerging evidence suggests that tumor-derived lactate regulates the pro-inflammatory response of monocyte and macrophage (Loftus and Finlay, 2016). The prominent heterogeneous immune cells in TME are macrophages, whose phenotypes are modulated by distinct signals in TME to exert significant effects on tumor dissemination (Colegio et al., 2014). Macrophages are composed primarily of classically activated (M1) and alternatively activated (M2) macrophages, with the latter frequently being displayed in pro-malignancy activity (Chen and Bonaldo, 2013). Indeed, there is growing evidence that acidification decreases the expression of CCL2, IL-6, and iNOS in M1 macrophages while increasing the expression of markers in M2 macrophages within the tumor milieu. It has been recommended that the metabolic lactate, rather than a decreased pH, causes macrophages to polarize in an M2-like manner (Su et al., 2014). Tumor-derived lactate has also been shown to induce M2-like polarization in THP1 and LPS-activated human monocytes (Ye et al., 2018). Growing evidence suggests that tumor-derived lactate educates macrophages to become functional tumor-associated macrophages (TAMs), the largest population of stromal cells that inhibit immune responses and promote tumor evolution, leading to poor clinical outcomes (Chen et al., 2011).

Lactate also inhibits the production of TNFαand IL-6 in LPS-stimulated macrophages. According to research, lactate signals by binding to its receptor G protein-couple receptor 81 (GPR81). K Yang et al. demonstrated that lactate suppressed LPS-stimulated NF-kB and YAP activation and nuclear translocation through its receptor GPR81-mediated AMPK/LATS activation (Yang et al., 2020). It also modulates macrophage phenotype by regulating the hypoxia-inducible factor 1α (HIF1α)–vascular endothelial cell growth factor (VEGF) signaling pathway (Colegio et al., 2014) and the secretion of ARG1. Furthermore, lactyl groups produced from lactate may contribute to post-translational modifications of histone proteins, resulting in increased expression of M2 marker genes such as ARG1 and IL-6.

## Targeting Lactate Metabolism and Signaling Effectively Inhibits Tumor Progression

### Regulation of Lactate Related Signaling Pathway

Understanding the role of lactate tumor dissemination in dampening lactate homeostasis is a promising link for improving tumor treatments. Interventions aimed at the activity or expression of the molecules involved in the deregulated metabolic pathways of glycolysis and glutaminolysis will inevitably inhibit lactate production and release. For example, in preclinical cancer models, HIF1-dependent signaling, aberrant MYC expression, and activated PI3K signaling are all favorable events for glutaminolysis that have been reported to be potentially targetable to achieve positive results (Rey et al., 2017; Whitfield et al., 2017; Janku et al., 2018). Nonetheless, since these molecular players control multiple signaling and metabolic events, clinicians face significant challenges in achieving the expected efficacy of impairing lactate homeostasis.

Considering the effects of extracellular acidification on tumor evolution, regulating the pH within TME is regarded as a valid measure to improve anti-tumor efficacy. pH regulators (MCTs, Na^+^/H^+^ exchangers and Na^+^/HCO_3_^−^ co-transporters, carbonic anhydrases, and anion exchangers) were successfully targeted with antibodies and molecules. From another point of view, neutralizing the acidity of TME could restore immune responses to immunotherapy induced by checkpoint inhibitors such as antibodies against programmed cell death protein 1 (PD-1) and cytotoxic T lymphocyte-associated antigen 4 (CTLA-4) (Balgi et al., 2011). We have a reasonable hypothesis that the metabolic couplings in the tumor milieu provide a unique opportunity to develop drugs that target tumor metabolism. Furthermore, this provides new sights for improving the anti-tumor efficacy of immunotherapy by restoring the metabolic fitness of the host immune system.

### Inhibitors Hindering the Function of the Lactate Transporter Effectively Contribute to Tumor Therapy

Interfering with intercellular lactate transport by targeting MCTs has significant effects. MCT1-inhibitors impair lactate transport, resulting in a metabolic switch from lactate that fuels OXPHOS to glycolysis, indirectly eliminating tumor cells in the hypoxic region via glucose deprivation. On the one hand, MCT1 inhibitors may influence lactate influx, which initially benefits cancer cells by adapting to glucose depletion. On the other hand, targeting MCT1 alleviates resistance to anti-angiogenic therapy. For instance, AR-C155858 is an MCT inhibitor that targets MCT1 and MCT2, whereas SR13800 is an MCT inhibitor that only targets MCT1. Besides, promising results have been acquired with the AstraZeneca compound AZ3965 (Polanski et al., 2014), an inhibitor that targets both MCT1 and MCT2 but with a 6-fold stronger selectivity on MCT1. Moreover, a small molecule is known as α-cyano-4-hydroxycinnamate (CHC) effectively inhibits tumor initiation, progression, and metastasis in glioblastoma (Mathupala et al., 2004). In murine cancer models treated with CHC, decreased tumor evolution and necrosis in the core region were consistently observed.

### Targeting Key Enzymes in the Lactate Decomposition and Synthesis Pathway Exerts Strong Anti-Tumor Effects

According to growing evidence, targeting lactate oxidase (LOX) may supplement traditional treatment and improve therapeutic efficacy. Several studies have reported nanoparticles loaded with LOX in treating malignant diseases. The Warburg-dependent tumor cells require a lot of glucose and produce excessive lactate in TME. TME lactate and acidosis can impair immune surveillance by negatively modulating tumor-infiltrating immune cells (Colegio et al., 2014; Hinshaw and Shevde, 2019; Pucino et al., 2019). LOX can alleviate acidification and consume lactate in the TME, activate tumor immune response and reshape TME by facilitating DC cell generation, M2 repolarization, reducing immunosuppressive cells (Tregs) infiltration, and enhancing immune effector cells (NKs, CTLs) functions.

Meanwhile, excess H_2_O_2_ kills tumor cells due to its high oxidative capacity and has synergistic effects when combined with immunotherapy. In recent decades, an increasing number of nanomaterials have been synthesized to exert anti-tumor effects (Alexis et al., 2008; Phan et al., 2009). Multiple novel tumor treatments derived from nanomedicine have opened new avenues for treating malignant tumors. For example, methylcellulose (MC) hydrogel loaded with lactate oxidase (LOX) (MC-LOX) was observed to contribute to M2 to M1 macrophages repolarization by consuming lactate in TME (Liao et al., 2019). Dendritic mesoporous silica nanoparticles (ODMSNs) loaded with LOX have been shown to suppress tumor angiogenesis by consuming more than 99.9% lactate in TME (Tang et al., 2020).

Targeting LDHA activation has been shown to help inhibit the conversion of pyruvate to lactate. Given that LDHA is the predominant isoform found in tumors, a subset of compounds targeting LDHA has emerged and been validated in preclinical studies. Compounds such as gossypol derivative (AT-101), FX-11, N-hydroxy indole-based, and galloflavin successfully inhibit LDHA from performing anti-tumor effects in pancreatic ductal adenocarcinoma and cervical cancer (Granchi et al., 2011; Dorneburg et al., 2018).

Even though current research has confirmed that highly produced and accumulated lactate in the TME could be regarded as highly potential anti-tumor targets, we face great challenges in translating this finding into clinical treatment. To improve patient prognosis by regulating tumor metabolism and exploring more effective novel combination therapies, we must first determine how lactate affects host immune responses and chemo-radiotherapy resistance.

### The Significance of Modulating Lactate in Improving Tumor Immunotherapy

Immunotherapy is broadly classified as immune checkpoint blockade (ICB), chimeric antigen receptor T cells therapy (CAR-T), and tumor vaccines, all of which effectively eliminate tumor cells by activating the host immune response. Immune checkpoints have been shown to have important effects on self-tolerance in the immune system, with inhibitory checkpoints being a possible target spot. ICB improves T lymphocyte function by intercepting co-inhibitory molecules and reactivating host immune responses (Xia et al., 2017). These two major checkpoints involve an interaction between the programmed cell death protein 1 (PD-1) and the cytotoxic T lymphocyte antigen 4 (CTLA-4) on T cells and their ligands, PD-L1 and CD80/CD86, respectively, are detected on immune cells under physiological conditions. The use of fusion proteins and antibodies against CTLA-4, PD-1, and PD-L1 in the treatment of malignancies has been extremely successful. Additionally, T cells express chimeric antigen receptors (CAR), which specifically capture antigens on tumor surfaces to eliminate tumor cells (Pettitt et al., 2018). Furthermore, tumor vaccines are novel methods to elicit antigen-specific immune responses (Vansteenkiste et al., 2013; Milani et al., 2014; Newick et al., 2017).

Even though immunotherapy has demonstrated obvious efficacy, there is still a subset of defects that must be addressed, such as narrow anti-neoplastic spectrum, severe adverse effects, and limited efficacy, emphasizing the importance of modulating tumor metabolism to alter the immune state of TME. Determining the immunotherapy resistance mechanism is critical and exploring combined therapies to boost anti-tumor immunity and long-term responses. It has been reported that the resistance to tumor immunotherapy is possibly caused by ineffective T cell activation and infiltration in the immunosuppressive TME. Furthermore, numerous studies illustrated multiple elements in the reprogrammed TME that lead to immune tolerance, like accumulated lactate and highly-expressed co-inhibitory molecules in TME. We highlighted how the lactate concentration in TME reacted in tumor immunotherapy resistance and summarized the relevant pathways that could be targeted in the combined therapy.

Due to the Warburg effect, excess tumor-derived lactate accumulates in TME, along with CO_2_ and many other metabolites. Studies have reported that high lactate concentrations dampen the functions of human CTLs to proliferate and produce cytokines (Garon et al., 2015). Also, lactate-induced acidosis could reduce arginine levels in the TME by facilitating ARG1 expression in macrophages, which ultimately suppressed the activation, proliferation, and activities of human CD8^+^T cells (Mendler et al., 2012). Additionally, intracellular lactate inhibits T-cell glycolysis via inhibiting PI3K/AKT/mTOR signaling pathway (Pilon-Thomas et al., 2016). Na Li et al. observed that deleting the N6-methylation of adenosine (m6^A^) demethylase Alkbh5 sensitized tumors to cancer immunotherapy in well-established ICB mouse cancer models (Lia et al., 2020). Notably, Alkbh5 modulates Mct4/Slc16a3 expression and lactate content of TME and the composition of myeloid-derived suppressor cells (MDSCs) and tumor-infiltrating Tregs. Importantly, a small molecule Alkbh5 inhibitor improved the anti-tumor effects of immunotherapy. Above all, thoroughly analyzing the emerging co-activate components in TME (especially the tumor-derived lactate) is critical to overcoming the limitations of immunotherapy. Combining immunotherapy with lactate targeting in TME could be a promising strategy, and exploring them is undeniably important in improving the prognosis of cancer patients.

## Summary and Outlook

This review first discussed the Warburg effect, a characteristic way tumor cells survive. Simultaneously, we summarized the generation, transport, and shuttling of the end-product lactate, as well as the specific role lactate played in tumor progression. Furthermore, we explored how lactate facilitates angiogenesis, serves as an energy source, mediates epigenetic modification, modulates an immunosuppressive TME, and influences tumor therapy’s efficacy. We extensively complement recent advances in histone modification, focusing on the phenomenon that lactate modifies histones to regulate macrophage polarization and tumor immunity, which is one of the highlights of this manuscript. Ultimately, we summarized potential anti-tumor strategies that target lactate metabolism and signaling. We elucidated that applying lactate oxidase (LOX) in combination with nanomedicine to consume lactate in TME to exert anti-tumor effects has also demonstrated obvious potential, which is another high point of this manuscript.

Several studies have shown that the metabolite lactate may be a significant obstacle to tumor eradication. Comprehensive studies are expected to delineate further the detailed downstream signals triggered by lactate, potentially providing therapeutic targets for cancer treatments. Emerging evidence suggests that excessive lactate in TME may be a defining feature of various malignancies, providing a neoteric mode of epigenetic and metabolic aberration in oncogenesis. Based on these findings, the importance of modulating tumor metabolism in cancer treatment has renewed researcher’s interests in decades.

The rising incidence and mortality rates of malignant tumors pose a serious threat to public health worldwide, necessitating the development of safe and effective treatment. Current tumor treatments include surgical resection, chemotherapy, radiotherapy, and gene-targeted therapy, which have failed to provide satisfactory efficacy for patient’s prognosis. These clinical discoveries inspire a thorough understanding of multiple components in TME and their complex functions, especially couplings associated with lactate, providing new possibilities for exploring broader and more effective combined therapies to compete to combat malignancies. Despite these significant obstacles in anti-tumor therapy, it has been proved that natural, induced, and engineered immune responses to tumors can effectively improve clinical efficacy, especially in certain malignancies.

Therapeutic strategies aimed at certain metabolic pathways are becoming more effective and convincing. Current tumor treatments in combination with compounds that target the lactate signal to achieve better anti-tumor effects also hold promise. More comprehensive studies focusing on tumor metabolic characteristics, lactate metabolism signaling pathway, and interactions between lactate and other components in TME are needed to complete our understanding of the environment required for tumor progression, which will contribute to the synthesis of novel drugs and therapeutic patterns with higher efficacy and fewer side effects.

## References

[B1] Alexis F. Rhee J.-W. Richie J. P. Radovic-Moreno A. F. Langer R. Farokhzad O. C. (2008). New Frontiers in Nanotechnology for Cancer Treatment. Urol. Oncol. Semin. Original Invest. 26, 74–85. 10.1016/j.urolonc.2007.03.017 18190835

[B2] Almand B. Resser J. R. Lindman B. Nadaf S. Clark J. I. Kwon E. D. (2000). Clinical Significance of Defective Dendritic Cell Differentiation in Cancer. Clin. Cancer Res. 6, 1755–1766. 10.1159/000007283 10815894

[B3] André P. Denis C. Soulas C. Bourbon-Caillet C. Lopez J. Arnoux T. (2018). Anti-NKG2A mAb Is a Checkpoint Inhibitor that Promotes Anti-tumor Immunity by Unleashing Both T and NK Cells. Cell 175, 1731–e13. 10.1016/j.cell.2018.10.014 30503213 PMC6292840

[B4] Arseneault R. Chien A. Newington J. T. Rappon T. Harris R. Cumming R. C. (2013). Attenuation of LDHA Expression in Cancer Cells Leads to Redox-dependent Alterations in Cytoskeletal Structure and Cell Migration. Cancer Lett. 338, 255–266. 10.1016/j.canlet.2013.03.034 23583676

[B5] Baek G. Tse Y. F. Hu Z. Cox D. Buboltz N. McCue P. (2014). MCT4 Defines a Glycolytic Subtype of Pancreatic Cancer with Poor Prognosis and Unique Metabolic Dependencies. Cel Rep. 9, 2233–2249. 10.1016/j.celrep.2014.11.025 25497091

[B6] Balgi A. D. Diering G. H. Donohue E. Lam K. K. Fonseca B. D. Zimmerman C. (2011). Regulation of mTORC1 Signaling by pH. PLoS ONE 6, e21549. 10.1371/journal.pone.0021549 21738705 PMC3126813

[B7] Banchereau J. Steinman R. M. (1998). Dendritic Cells and the Control of Immunity. Nature 392, 245–252. 10.1038/32588 9521319

[B8] Bell D. Young J. W. Banchereau J. (1999). Dendritic Cells. Adv. Immunol. 72, 255–324. 10.1016/s0065-2776(08)60023-1 10361578

[B9] Bogunovic D. O'Neill D. W. Belitskaya-Levy I. Vacic V. Yu Y. L. Adams S. (2009). Immune Profile and Mitotic index of Metastatic Melanoma Lesions Enhance Clinical Staging in Predicting Patient Survival. Proc. Natl. Acad. Sci. USA 106, 20429–20434. 10.1073/pnas.0905139106 19915147 PMC2787158

[B10] Brand A. Singer K. Koehl G. E. Kolitzus M. Schoenhammer G. Thiel A. (2016). LDHA-associated Lactic Acid Production Blunts Tumor Immunosurveillance by T and NK Cells. Cel Metab. 24, 657–671. 10.1016/j.cmet.2016.08.011 27641098

[B11] Cascone T. McKenzie J. A. Mbofung R. M. Punt S. Wang Z. Xu C. (2018). Increased Tumor Glycolysis Characterizes Immune Resistance to Adoptive T Cell Therapy. Cel Metab. 27, 977–987. 10.1016/j.cmet.2018.02.024 PMC5932208 29628419

[B12] Certo M. Marone G. de Paulis A. Mauro C. Pucino V. (2019). Lactate: Fueling the Fire Starter. Wiley Interdiscip. Rev. Syst. Biol. Med. 12, e1474. 10.1002/wsbm.1474 31840439 PMC7187281

[B13] Chang C.-H. Qiu J. O’Sullivan D. Buck M. D. Noguchi T. Curtis J. D. (2015). Metabolic Competition in the Tumor Microenvironment Is a Driver of Cancer Progression. Cell 162, 1229–1241. 10.1016/j.cell.2015.08.016 26321679 PMC4864363

[B14] Chen J. Yao Y. Gong C. Yu F. Su S. Chen J. (2011). CCL18 from Tumor-Associated Macrophages Promotes Breast Cancer Metastasis via PITPNM3. Cancer Cell 19 (4), 541–555. 10.1016/j.ccr.2011.02.006 21481794 PMC3107500

[B15] Chen P. Bonaldo P. (2013). Role of Macrophage Polarization in Tumor Angiogenesis and Vessel Normalization. Int. Rev. Cel Mol Biol 301, 1–35. 10.1016/b978-0-12-407704-1.00001-4 23317816

[B16] Colegio O. R. Chu N.-Q. Szabo A. L. Chu T. Rhebergen A. M. Jairam V. (2014). Functional Polarization of Tumour-Associated Macrophages by Tumour-Derived Lactic Acid. Nature 513 (7519), 559–563. 10.1038/nature13490 25043024 PMC4301845

[B17] Cong J. Wang X. Zheng X. Wang D. Fu B. Sun R. (2018). Dysfunction of Natural Killer Cells by FBP1-Induced Inhibition of Glycolysis during Lung Cancer Progression. Cel Metab. 28, 243–255. 10.1016/j.cmet.2018.06.021 30033198

[B18] Connor H. Woods H. F. Ledingham J. G. (2017). Comparison of the Kinetics and Utilisation of D(-)-and L(+)-sodium Lactate in normal Man. Ann. Nutr. Metab. 27, 481–487. 10.1159/000176723 6651225

[B19] Crabtree H. G. (1929). Observations on the Carbohydrate Metabolism of Tumours. Biochem. J. 23, 536–545. 10.1042/bj0230536 16744238 PMC1254097

[B20] Dennison J. B. Molina J. R. Mitra S. González-Angulo A. M. Balko J. M. Kuba M. G. (2013). Lactate Dehydrogenase B: A Metabolic Marker of Response to Neoadjuvant Chemotherapy in Breast Cancer. Clin. Cancer Res. 19, 3703–3713. 10.1158/1078-0432.ccr-13-0623 23697991 PMC3727144

[B21] Di Vito C. Mikulak J. Zaghi E. Pesce S. Marcenaro E. Mavilio D. (2019). NK Cells to Cure Cancer. Semin. Immunol. 41, 101272. 10.1016/j.smim.2019.03.004 31085114

[B22] Dorneburg C. Fischer M. Barth T. F. E. Mueller-Klieser W. Hero B. Gecht J. (2018). LDHA in Neuroblastoma Is Associated with Poor Outcome and its Depletion Decreases Neuroblastoma Growth Independent of Aerobic Glycolysis. Clin. Cancer Res. 24, 5772–5783. 10.1158/1078-0432.ccr-17-2578 29925504

[B23] El-Kenawi A. Ibrahim-Hashim A. A. Luddy K. A. Pilon-Thomas S. A. Gatenby R. A. Gillies R. A., (2015). Extracellular Acidosis Alters Polarization of Macrophages. Cancer Res. 75, 3213. 10.1158/1538-7445.AM2015-3213

[B24] Faubert B. Li K. Y. Cai L. Hensley C. T. Kim J. Zacharias L. G. (2017). Lactate Metabolism in Human Lung Tumors. Cell 171, 358–371. 10.1016/j.cell.2017.09.019 28985563 PMC5684706

[B25] Galván-Peña S. O’Neill L. A. J. (2014). Metabolic Reprograming in Macrophage Polarization. Front. Immunol. 5, 420. 10.3389/fimmu.2014.00420 25228902 PMC4151090

[B26] Garcia C. K. Goldstein J. L. Pathak R. K. Anderson R. G. W. Brown M. S. (1994). Molecular Characterization of a Membrane Transporter for Lactate, Pyruvate, and Other Monocarboxylates: Implications for the Cori Cycle. Cell 76, 865–873. 10.1016/0092-8674(94)90361-1 8124722

[B27] Garon E. B. Rizvi N. A. Hui R. Leighl N. Balmanoukian A. S. Eder J. P. (2015). Pembrolizumab for the Treatment of Non-small-cell Lung Cancer. N. Engl. J. Med. 372, 2018–2028. 10.1056/nejmoa1501824 25891174

[B28] Girgis H. Masui O. White N. M. Scorilas A. Rotondo F. Seivwright A. (2014). Lactate Dehydrogenase A Is a Potential Prognostic Marker in clear Cell Renal Cell Carcinoma. Mol. Cancer 13, 101. 10.1186/1476-4598-13-101 24885701 PMC4022787

[B29] Gouirand V. Guillaumond F. Vasseur S. (2018). Influence of the Tumor Microenvironment on Cancer Cells Metabolic Reprogramming. Front. Oncol. 8, 117. 10.3389/fonc.2018.00117 29725585 PMC5917075

[B30] Granchi C. Roy S. Giacomelli C. Macchia M. Tuccinardi T. Martinelli A. (2011). Discovery of N-Hydroxyindole-Based Inhibitors of Human Lactate Dehydrogenase Isoform A(LDH-A) as Starvation Agents Againstcancer Cells. J. Med. Chem. 54, 1599–1612. 10.1021/jm101007q 21332213

[B31] Habif G. Crinier A. André P. Vivier E. Narni-Mancinelli E. (2019). Targeting Natural Killer Cells in Solid Tumors. Cell Mol Immunol 16, 415–422. 10.1038/s41423-019-0224-2 30911118 PMC6474204

[B32] Harmon C. Robinson M. W. Hand F. Almuaili D. Mentor K. Houlihan D. D. (2019). Lactate-Mediated Acidification of Tumor Microenvironment Induces Apoptosis of Liver-Resident NK Cells in Colorectal Liver Metastasis. Cancer Immunol. Res. 7, 335–346. 10.1158/2326-6066.cir-18-0481 30563827

[B33] Hinshaw D. C. Shevde L. A. (2019). The Tumor Microenvironment Innately Modulates Cancer Progression. Cancer Res. 79, 4557–4566. 10.1158/0008-5472.can-18-3962 31350295 PMC6744958

[B34] Horikawa N. Abiko K. Matsumura N. Hamanishi J. Baba T. Yamaguchi K. (2017). Expression of Vascular Endothelial Growth Factor in Ovarian Cancer Inhibits Tumor Immunity through the Accumulation of Myeloid-Derived Suppressor Cells. Clin. Cancer Res. 23, 587–599. 10.1158/1078-0432.ccr-16-0387 27401249

[B35] Huang H. Sabari B. R. Garcia B. A. Allis C. D. Zhao Y. (2014). SnapShot: Histone Modifications. Cell 159, 458. 10.1016/j.cell.2014.09.037 25303536 PMC4324475

[B36] Hui S. Ghergurovich J. M. Morscher R. J. Jang C. Teng X. Lu W. (2017). Glucose Feeds the TCA Cycle via Circulating Lactate. Nature 551, 115–118. 10.1038/nature24057 29045397 PMC5898814

[B37] Husain Z. Huang Y. Seth P. Sukhatme V. P. (2013). Tumor-derived Lactate Modifies Antitumor Immune Response: Effect on Myeloid-Derived Suppressor Cells and NK Cells. J.I. 191, 1486–1495. 10.4049/jimmunol.1202702 23817426

[B38] Ippolito L. Morandi A. Giannoni E. Chiarugi P. (2019). Lactate: A Metabolic Driver in the Tumour Landscape. Trends Biochem. Sci. 44, 153–166. 10.1016/j.tibs.2018.10.011 30473428

[B39] Janku F. Yap T. A. Meric-Bernstam F. (2018). Targeting the PI3K Pathway in Cancer: Are We Making Headway? Nat. Rev. Clin. Oncol. 15, 273–291. 10.1038/nrclinonc.2018.28 29508857

[B40] Jiang F. Ma S. Xue Y. Hou J. Zhang Y. (2016). LDH-A Promotes Malignant Progression via Activation of Epithelial-To-Mesenchymal Transition and Conferring Stemness in Muscle-Invasive Bladder Cancer. Biochem. Biophysical Res. Commun. 469, 985–992. 10.1016/j.bbrc.2015.12.078 26721441

[B41] Jing H. Lin H. (2015). Sirtuins in Epigenetic Regulation. Chem. Rev. 115, 2350–2375. 10.1021/cr500457h 25804908 PMC4610301

[B42] Jones R. Morris M. (2016). Monocarboxylate Transporters: Therapeutic Targets and Prognostic Factors in Disease. Clin. Pharmacol. Ther. 100, 454–463. 10.1002/cpt.418 27351344 PMC5533588

[B43] Konjevi´c G. M. Vuleti´c A. M. Mirjaˇci´c Martinovi´c K. M. Larsen A. K. Juriši´c V. B. (2019). The Role of Cytokines in the Regulation of NK Cells in the Tumor Environment. Cytokine 117, 30–40. 10.1016/j.cyto.2019.02.00 30784898

[B44] Kouzarides T. (2007). Chromatin Modifications and Their Function. Cell 128, 693–705. 10.1016/j.cell.2007.02.005 17320507

[B45] Lee K. K. Workman J. L. (2007). Histone Acetyltransferase Complexes: One Size Doesn't Fit All. Nat. Rev. Mol. Cel Biol. 8, 284–295. 10.1038/nrm2145 17380162

[B46] Lewis P. W. Müller M. M. Koletsky M. S. Cordero F. Lin S. Banaszynski L. A. (2013). Inhibition of PRC2 Activity by a Gain-Of-Function H3 Mutation Found in Pediatric Glioblastoma. Science 340, 857–861. 10.1126/science.1232245 23539183 PMC3951439

[B47] Lia Na. Kanga Y. Wang L. Huff S. Tang R. Hui H. (2020). ALKBH5 Regulates Anti–PD-1 Therapy Response by Modulating Lactate and Suppressive Immune Cell Accumulation in Tumor Microenvironment. Proc. Natl. Acad. Sci. 117 (33), 201918986. 10.1073/pnas.1918986117 PMC7443867 32747553

[B48] Liao Z.-X. Fa Y.-C. Kempson I. M. Tseng S.-J. (2019). Repolarization of M2 to M1 Macrophages Triggered by Lactate Oxidase Released from Methylcellulose Hydrogel. Bioconjug. Chem. 30, 2697–2702. 10.1021/acs.bioconjchem.9b00618 31532192

[B49] Liu X. Yang Z. Chen Z. Chen R. Zhao D. Zhou Y. (2015). Effects of the Suppression of Lactate Dehydrogenase A on the Growth and Invasion of Human Gastric Cancer Cells. Oncol. Rep. 33, 157–162. 10.3892/or.2014.3600 25394466

[B50] Loftus R. M. Finlay D. K. (2016). Immunometabolism: Cellular Metabolism Turns Immune Regulator. J. Biol. Chem. 291 (1), 1–10. 10.1074/jbc.r115.693903 26534957 PMC4697146

[B51] Lyssiotis C. A. Kimmelman A. C. (2017). Metabolic Interactions in the Tumor Microenvironment. Trends Cel Biol. 27, 863–875. 10.1016/j.tcb.2017.06.003 PMC5814137 28734735

[B52] Macintyre A. N. Gerriets V. A. Nichols A. G. Michalek R. D. Rudolph M. C. Deoliveira D. (2014). The Glucose Transporter Glut1 Is Selectively Essential for CD4 T Cell Activation and Effector Function. Cel Metab. 20, 61–72. 10.1016/j.cmet.2014.05.004 PMC4079750 24930970

[B53] MacIver N. J. Jacobs S. R. Wieman H. L. Wofford J. A. Coloff J. L. Rathmell J. C. (2008). Glucose Metabolism in Lymphocytes Is a Regulated Process with Significant Effects on Immune Cell Function and Survival. J. Leukoc. Biol. 84, 949–957. 10.1189/jlb.0108024 18577716 PMC2638731

[B54] Mah A. Y. Cooper M. A. (2016). Metabolic Regulation of Natural Killer Cell IFN-γ Production. Crit. Rev. Immunol. 36, 131–147. 10.1615/critrevimmunol.2016017387 27910764 PMC5335907

[B55] Marchiq I. Pouysségur J. (2016). Hypoxia, Cancer Metabolism and the Therapeutic Benefit of Targeting lactate/H+ Symporters. J. Mol. Med. 94, 155–171. 10.1007/s00109-015-1307-x 26099350 PMC4762928

[B56] Markert C. L. Shaklee J. B. Whitt G. S. (1975). Evolution of a Gene. Science 189, 102–114. 10.1126/science.1138367 1138367

[B57] Marzi S. J. Leung S. K. Ribarska T. Hannon E. Smith A. R. Pishva E. (2018). A Histone Acetylome-wide Association Study of Alzheimer's Disease Identifies Disease-Associated H3K27ac Differences in the Entorhinal Cortex. Nat. Neurosci. 21, 1618–1627. 10.1038/s41593-018-0253-7 30349106

[B58] Mathupala S. P. Parajuli P. Sloan A. E. (2004). Silencing of Monocarboxylate Transporters via Small Interfering Ribonucleic Acid Inhibits Glycolysis and Induces Cell Death in Malignant Glioma: an *In Vitro* Study. Neurosurgery 55, 1410–1419. 10.1227/01.neu.0000143034.62913.59 15574223

[B59] Mendler A. N. Hu B. Prinz P. U. Kreutz M. Gottfried E. Noessner E. (2012). Tumor Lactic Acidosis Suppresses CTL Function by Inhibition of P38 and JNK/c-Jun Activation. Int. J. Cancer 131, 633–640. 10.1002/ijc.26410 21898391

[B60] Merezhinskaya N. Ogunwuyi S. A. Mullick F. G. Fishbein W. N. (2004). Presence and Localization of Three Lactic Acid Transporters (MCT1, −2, and −4) in Separated Human Granulocytes, Lymphocytes, and Monocytes. J. Histochem. Cytochem. 52, 1483–1493. 10.1369/jhc.4a6306.2004 15505343 PMC3957819

[B61] Milani A. Sangiolo D. Aglietta M. Valabrega G. (2014). Recent Advances in the Development of Breast Cancer Vaccines. Breast Cancer (Dove Med. Press. 6, 159–168. 10.2147/BCTT.S38428 25339848 PMC4204811

[B62] Moellering R. E. Cravatt B. F. (2013). Functional Lysine Modification by an Intrinsically Reactive Primary Glycolytic Metabolite. Science 341, 549–553. 10.1126/science.1238327 23908237 PMC4005992

[B63] Nayyar G. Chu Y. Cairo M. S. (2019). Overcoming Resistance to Natural Killer Cell Based Immunotherapies for Solid Tumors. Front. Oncol. 9, 51. 10.3389/fonc.2019.00051 30805309 PMC6378304

[B64] Newick K. O'Brien S. Moon E. Albelda S. M. (2017). CAR T Cell Therapy for Solid Tumors. Annu. Rev. Med. 68, 139–152. 10.1146/annurev-med-062315-120245 27860544

[B65] Orsini E. Guarini A. Chiaretti S. Mauro F. R. Foa R. (2003). The Circulating Dendritic Cell Compartment in Patients with Chronic Lymphocytic Leukemia Is Severely Defective and Unable to Stimulate an Effective T-Cell Response. Cancer Res. 63, 4497–4506. 10.1097/00002820-200308000-00012 12907623

[B66] Patel B. B. Ackerstaff E. Serganova I. S. Kerrigan J. E. Blasberg R. G. Koutcher J. A. (2017). Tumor Stroma Interaction Is Mediated by Monocarboxylate Metabolism. Exp. Cel Res. 352, 20–33. 10.1016/j.yexcr.2017.01.013 PMC5476446 28132882

[B67] Pavlova N. N. Thompson C. B. (2016). The Emerging Hallmarks of Cancer Metabolism. Cel Metab. 23, 27–47. 10.1016/j.cmet.2015.12.006 PMC4715268 26771115

[B68] Petrova V. Annicchiarico-Petruzzelli M. Melino G. Amelio I. (2018). The Hypoxic Tumour Microenvironment. Oncogenesis 7, 10. 10.1038/s41389-017-0011-9 29362402 PMC5833859

[B69] Pettitt D. Arshad Z. Smith J. Stanic T. Holländer G. Brindley D. (2018). CAR-T Cells: A Systematic Review and Mixed Methods Analysis of the Clinical Trial Landscape. Mol. Ther. 26, 342–353. 10.1016/j.ymthe.2017.10.019 29248427 PMC5835018

[B70] Phan J. H. Moffitt R. A. Stokes T. H. Liu J. Young A. N. Nie S. (2009). Convergence of Biomarkers, Bioinformatics and Nanotechnology for Individualized Cancer Treatment. Trends Biotechnol. 27, 350–358. 10.1016/j.tibtech.2009.02.010 19409634 PMC3779321

[B71] Pilon-Thomas S. Kodumudi K. N. El-Kenawi A. E. Russell S. Weber A. M. Luddy K. (2016). Neutralization of Tumor Acidity Improves Antitumor Responses to Immunotherapy. Cancer Res. 76, 1381–1390. 10.1158/0008-5472.can-15-1743 26719539 PMC4829106

[B72] Polanski R. Hodgkinson C. L. Fusi A. Nonaka D. Priest L. Kelly P. (2014). Activity of the Monocarboxylate Transporter 1 Inhibitor AZD3965 in Small Cell Lung Cancer. Clin. Cancer Res. 20, 926–937. 10.1158/1078-0432.CCR-13-2270 24277449 PMC3929348

[B73] Pucino V. Bombardieri M. Pitzalis C. Mauro C. (2017). Lactate at the Crossroads of Metabolism, Inflammation, and Autoimmunity. Eur. J. Immunol. 47, 14–21. 10.1002/eji.201646477 27883186

[B74] Pucino V. Certo M. Bulusu V. Cucchi D. Goldmann K. Pontarini E. (2019). Lactate Buildup at the Site of Chronic Inflammation Promotes Disease by Inducing CD4+ T Cell Metabolic Rewiring. Cel Metab. 30, 1055–1074. 10.1016/j.cmet.2019.10.004 PMC6899510 31708446

[B75] Rattigan Y. I. Patel B. B. Ackerstaff E. Sukenick G. Koutcher J. A. Glod J. W. (2012). Lactate Is a Mediator of Metabolic Cooperation between Stromal Carcinoma Associated Fibroblasts and Glycolytic Tumor Cells in the Tumor Microenvironment. Exp. Cel Res. 318, 326–335. 10.1016/j.yexcr.2011.11.014 PMC3402174 22178238

[B76] Renner K. Singer K. Koehl G. E. Geissler E. K. Peter K. Siska P. J. (2017). Metabolic Hallmarks of Tumor and Immune Cells in the Tumor Microenvironment. Front. Immunol. 8, 248. 10.3389/fimmu.2017.00248 28337200 PMC5340776

[B77] Rey S. Schito L. Wouters B. G. Eliasof S. Kerbel R. S. (2017). Targeting Hypoxia-Inducible Factors for Antiangiogenic Cancer Therapy. Trends Cancer 3, 529–541. 10.1016/j.trecan.2017.05.002 28718406

[B78] Rivera L. B. Bergers G. (2015). Intertwined Regulation of Angiogenesis and Immunity by Myeloid Cells. Trends Immunol. 36, 240–249. 10.1016/j.it.2015.02.005 25770923 PMC4393787

[B79] Rivera L. B. Meyronet D. Hervieu V. Frederick M. J. Bergsland E. Bergers G. (2015). Intratumoral Myeloid Cells Regulate Responsiveness and Resistance to Antiangiogenic Therapy. Cel Rep. 11, 577–591. 10.1016/j.celrep.2015.03.055 PMC4438771 25892230

[B80] Ronan J. L. Wu W. Crabtree G. R. (2013). From Neural Development to Cognition: Unexpected Roles for Chromatin. Nat. Rev. Genet. 14, 347–359. 10.1038/nrg3413 23568486 PMC4010428

[B81] Sankar A. Lerdrup M. Manaf A. Johansen J. V. Gonzalez J. M. Borup R. (2020). KDM4A Regulates the Maternal-To-Zygotic Transition by Protecting Broad H3K4me3 Domains from H3K9me3 Invasion in Oocytes. Nat. Cel Biol 22, 380–388. 10.1038/s41556-020-0494-z PMC7212036 32231309

[B82] Shi Y. Du L. Lin L. Wang Y. (2017). Tumour-associated Mesenchymal Stem/stromal Cells: Emerging Therapeutic Targets. Nat. Rev. Drug Discov. 16, 35–52. 10.1038/nrd.2016.193 27811929

[B83] Sonveaux P. Copetti T. De Saedeleer C. J. Végran F. Verrax J. Kennedy K. M. (2012). Targeting the Lactate Transporter MCT1 in Endothelial Cells Inhibits Lactate-Induced HIF-1 Activation and Tumor Angiogenesis. PLoS One 7, e33418. 10.1371/journal.pone.0033418 22428047 PMC3302812

[B84] Stojanovic A. Correia M. P. Cerwenka A. (2013). Shaping of NK Cell Responses by the Tumor Microenvironment. Cancer Microenvironment 6, 135–146. 10.1007/s12307-012-0125-8 23242671 PMC3717064

[B86] Su S. Liu Q. Chen J. Chen J. Chen F. He C. (2014). A Positive Feedback Loop between Mesenchymal-like Cancer Cells and Macrophages Is Essential to Breast Cancer Metastasis. Cancer Cell 25 (5), 605–620. 10.1016/j.ccr.2014.03.021 24823638

[B87] Sutherland R. M. (1988). Cell and Environment Interactions in Tumor Microregions: The Multicell Spheroid Model. Science 240, 177–184. 10.1126/science.2451290 2451290

[B88] Tang J. Meka A. K. Theivendran S. Wang Y. Yang Y. Song H. (2020). Openwork@Dendritic Mesoporous Silica Nanoparticles for Lactate Depletion and Tumor Microenvironment Regulation. Angew. Chem. Int. Ed. 59, 22054–22062. 10.1002/anie.202001469 32705778

[B89] Tessarz P. Kouzarides T. (2014). Histone Core Modifications Regulating Nucleosome Structure and Dynamics. Nat. Rev. Mol. Cel Biol 15, 703–708. 10.1038/nrm3890 25315270

[B90] Trivedi B. Danforth W. H. (2016). Effect of pH on the Kinetics of Frog Muscle Phosphofructokinase. J. Biol. Chem. 241, 4110–4112. 10.1016/S0021-9258(18)99819-4 4224144

[B91] Valvona C. Fillmore H. (2018). Oxamate, but Not Selective Targeting of LDH-A, Inhibits Medulloblastoma Cell Glycolysis, Growth and Motility. Brain Sci. 8, 56. 10.3390/brainsci8040056 PMC5924392 29601482

[B92] Vansteenkiste J. Zielinski M. Linder A. Dahabreh J. Gonzalez E. E. Malinowski W. (2013). Adjuvant MAGE-A3 Immunotherapy in Resected Non-small-cell Lung Cancer: Phase II Randomized Study Results. Jco 31, 2396–2403. 10.1200/jco.2012.43.7103 23715567

[B93] Vaupel P. (2008). Hypoxia and Aggressive Tumor Phenotype: Implications for Therapy and Prognosis. Oncologist 13 Suppl 3 (Suppl. 3), 21–26. 10.1634/theoncologist.13-S3-21 18458121

[B94] Vaupel P. Multhoff G. (2016). A Metabolic Immune Checkpoint: Adenosine in Tumor Microenvironment. Front. Immunol. 7, 3329. 10.3389/fimmu.2016.00332 PMC5006596 27629240

[B95] Vaupel P. Mayer A. (2016). Hypoxia-driven Adenosine Accumulation: a Crucial Microenvironmental Factor Promoting Tumor Progression. Adv. Exp. Med. Biol. 876, 177–183. 10.1007/978-1-4939-3023-4_22 26782210

[B96] Wagner G. R. Payne R. M. (2013). Widespread and Enzyme-independent Nϵ-Acetylation and Nϵ-Succinylation of Proteins in the Chemical Conditions of the Mitochondrial Matrix*. J. Biol. Chem. 288, 29036–29045. 10.1074/jbc.m113.486753 23946487 PMC3790002

[B97] Walenta S. Schroeder T. Mueller-Klieser W. (2004). Lactate in Solid Malignant Tumors: Potential Basis of a Metabolic Classification in Clinical Oncology. Cmc 11, 2195–2204. 10.2174/0929867043364711 15279558

[B98] Warburg O. Posener K. Negelein E. (1924). Ueber den stoffwechsel der tumoren. Biochem. Z. 152, 319–3443.

[B99] Warburg O. (1925). The Metabolism of Carcinoma Cells. J. Cancer Res. 9, 148–163. 10.1158/jcr.1925.148

[B100] Weinert B. T. Moustafa T. Iesmantavicius V. Zechner R. Choudhary C. (2015). Analysis of Acetylation Stoichiometry Suggests that SIRT3 Repairs Nonenzymatic Acetylation Lesions. EMBO J. 34, 2620–2632. 10.15252/embj.201591271 26358839 PMC4641529

[B101] Weinert B. T. Iesmantavicius V. Moustafa T. Schölz C. Wagner S. A. Magnes C. (2014). Acetylation Dynamics and Stoichiometry in S Accharomyces Cerevisiae. Mol. Syst. Biol. 10, 716. 10.1002/msb.134766 24489116 PMC4023402

[B102] Whitfield J. R. Beaulieu M. E. Soucek L. (2017). Strategies to Inhibit Myc and Their Clinical Applicability. Front. Cel Dev. Biol 5, 10. 10.3389/fcell.2017.00010 PMC5322154 28280720

[B103] Xia A.-L. Wang X.-C. Lu Y.-J. Lu X.-J. Sun B. (2017). Chimeric-antigen Receptor T (CAR-T) Cell Therapy for Solid Tumors: Challenges and Opportunities. Oncotarget 8, 90521–90531. 10.18632/oncotarget.19361 29163850 PMC5685771

[B109] Yang K. Xu J. Fan M. Tu F. Wang X. Ha T. (2020). Lactate Suppresses Macrophage Pro-inflammatory Response to LPS -stimulation by Inhibition of YAP and NF-kB Activation via GPR81-Mediated Signaling. Front. Immunol. 11, 587913. 10.3389/fimmu.2020.587913 33123172 PMC7573489

[B104] Ye H. Zhou Q. Zheng S. Li G. Lin Q. Wei L. (2018). Tumor-associated Macrophages Promote Progression and the Warburg Effect via CCL18/NF-kB/VCAM-1 Pathway in Pancreatic Ductal Adenocarcinoma. Cell Death Dis 9, 453. 10.1038/s41419-018-0486-0 29670110 PMC5906621

[B105] Yu Jie. Chai Peiwei. Xie Minyue. Ge S. Ruan J. Fan X. (2021). Histone Lactylation Drives Oncogenesis by Facilitating M6 A Reader Protein YTHDF2 Expression in Ocular Melanoma. Genome Biol. 22, 85. 10.1186/s13059-021-02308-z 33726814 PMC7962360

[B106] Ždralevi´c M. Brand A. Di Ianni L. Dettmer K. Reinders J. Singer K. (2018). Double Genetic Disruption of Lactate dehydrogenasesA and B Is Required to Ablate the “Warburg Effect” Restricting Tumor Growth to Oxidative Metabolism. J. Biol. Chem. 293, 15947–15961. 10.1074/jbc.RA118.004180 30158244 PMC6187639

[B107] Zhang D. Tang Z. Huang H. Zhou G. Cui C. Weng Y. (2019). Metabolic Regulation of Gene Expression by Histone Lactylation. Nature 574, 575–580. 10.1038/s41586-019-1678-1 31645732 PMC6818755

[B108] Zhang Z. Li Y. Yan X. Song Q. Wang G. Hu Y. (2019). Pretreatment Lactate Dehydrogenase May Predict Outcome of Advanced Non Small‐cell Lung Cancer Patients Treated with Immune Checkpoint Inhibitors: A Meta‐analysis. Cancer Med. 8, 1467–1473. 10.1002/cam4.2024 30848091 PMC6488146

